# Detoxification of bilirubin and bile acids with intermittent coupled plasmafiltration and adsorption in liver failure (HERCOLE study)

**DOI:** 10.1007/s40620-020-00799-w

**Published:** 2020-07-24

**Authors:** Gabriele Donati, Andrea Angeletti, Lorenzo Gasperoni, Fabio Piscaglia, Anna Laura Croci Chiocchini, Anna Scrivo, Teresa Natali, Ines Ullo, Chiara Guglielmo, Patrizia Simoni, Rita Mancini, Luigi Bolondi, Gaetano La Manna

**Affiliations:** 1 Department of Experimental, Diagnostic and Specialty Medicine (DIMES), Nephrology, Dialysis and Renal Transplantation Unit, S. Orsola Hospital, University of Bologna, Via G. Massarenti 9 (Pad. 15), 40138 Bologna, Italy; 2 Internal Medicine Unit, S. Orsola Hospital, University of Bologna, Bologna, Italy; 3 Laboratory of Gastroenterology, S. Orsola Hospital, University of Bologna, Bologna, Italy; 4 Metropolitan Laboratory, S. Orsola Hospital, University of Bologna, Bologna, Italy

**Keywords:** Liver failure, Plasmafiltration, Adsorption, Liver detoxification, Hemofiltration

## Abstract

**Background:**

CPFA is an extracorporeal treatment used in severe sepsis to remove circulating proinflammatory cytokines. Limited evidence exists on the effectiveness of bilirubin adsorption by the hydrophobic styrenic resin, the distinctive part of CPFA. The aim of this study is to validate CPFA effectiveness in liver detoxification.

**Methods:**

In this prospective observational study, we enrolled patients with acute or acute-on-chronic liver failure (serum total bilirubin > 20 mg/dL or MELD Score > 20) hospitalized from June 2013 to November 2017. CPFA was performed using the Lynda (Bellco/MedTronic, Mirandola, Italy) or the Amplya (Bellco/MedTronic, Mirandola, Italy) machines. Anticoagulation was provided with unfractionated heparin or citrate. Bilirubin and bile acids reduction ratios per session (RRs) were the main parameters for hepatic detoxification.

**Results:**

Twelve patients with acute (n = 3) or acute-on-chronic (n = 9) liver failure were enrolled. Alcohol was the main cause of liver disease. Thirty-one CPFA treatments of 6 h each were performed, 19 with heparin and 12 with citrate. RRs was 28.8% (range 2.2–40.5) for total bilirubin, 32.7% (range 8.3–48.9) for direct bilirubin, 29.5% (range 6.5–65.4) for indirect bilirubin and 28.9% (16.7- 59.7) for bile acids. One patient received liver transplantation and 8/9 were alive at 1 year of follow-up. Three patients (25%) died: 2 during hospitalization and 1 for a cardiac event at 4 months of follow up with restored liver function.

**Conclusions:**

CPFA resulted to be effective in liver detoxification. Thus, it may be considered as a “bridge technique” both to the liver transplant and to the recovery of the basal liver function.

## Introduction

The search for an extracorporeal purification system for acute liver failure (ALF) or acute-on-chronic liver failure (AoCLF) is still a matter of debate. The pathophysiologic hypothesis informing the use of extracorporeal purification during ALF or AoCLF is the assumption that a vicious circle is initiated by the failing liver: during ALF or AoCLF toxins accumulate and cause direct damage to the liver itself, leading in turn to the faster accumulation of toxins [1–3]. Consequently, several efforts have been made to establish adequate blood purifications methods for the removal of hepatic toxins. The two largest randomized controlled trials, the HELIOS and RELIEF studies, failed to demonstrate the superiority of extracorporeal depurative treatment associated with standard medical therapy, compared to medical therapy alone, in terms of survival [4, 5]. However, a non-inferiority of the extracorporeal treatments was reported and the treatment with Prometheus was associated with higher survival in patients with MELD > 30 [4].

Despite the weak clinical evidence, artificial liver support systems appear to be safe and are normally characterized by the improvement of serum biochemical parameters, hemodynamic status, and hepatic encephalopathy [6]. Experiences with extracorporeal techniques for liver failure are currently limited and only one prospective randomized controlled trial, testing the effectiveness of high-volume plasma exchange in patients with ALF, has demonstrated that treatment with high-volume plasma exchange increases the liver transplant-free survival of patients affected by ALF [7].

The need for a safe and effective extracorporeal depurative treatment for patients with liver failure is testified by the widening gap between patients with ALF and AoCLF and those who receive a liver transplantation [8]. According to the National Center for Health Statistics [8], between 1999 and 2006 over 10,000 patients died due to ALF and only 3,000 patients affected by ALF were the on waiting list for orthotopic liver transplantation (OLT). Among patients suffering from end-stage liver failure, there were approximately 400,000 deaths over the same period and 50,000 patients were on the waiting list for OLT.

Since most of the toxins that accumulate during liver failure are bound to albumin, their removal still represents a technological challenge. The link between bilirubin and albumin is extremely effective (9.5 × 10^7^ M^−1^) [9] and a mere ninety-five millionth of a mole of bilirubin is able to saturate 50% of its available bonds on albumin. A removal system able to break the molecular bonds between albumin and toxic substances is still missing. Currently, a detoxification technique with adsorbent properties is the only available system to remove albumin-bound toxins. The adsorption consists in the consolidation on the surface of a solid material of molecules floating in a fluid after matching the fluid with the solid. The consolidation is due to weak molecular bonds, such as Van der Waals forces and hydrophobic bonds. The selectivity of adsorption occurs when albumin molecules easily pass through the sorbent, while the albumin carrying toxins is held into the pores. Hepatic toxins are often hydrophobic, with a molecular weight < 1000 Da (bilirubin = 406 Da, cholic acid = 283 Da, chenodeoxycholic acid = 272 Da). Relative protein binding is a physiological protective mechanism to prevent the free diffusion of bilirubin and bile acids with consequent toxicity in human tissues.

**Fig. 1:**
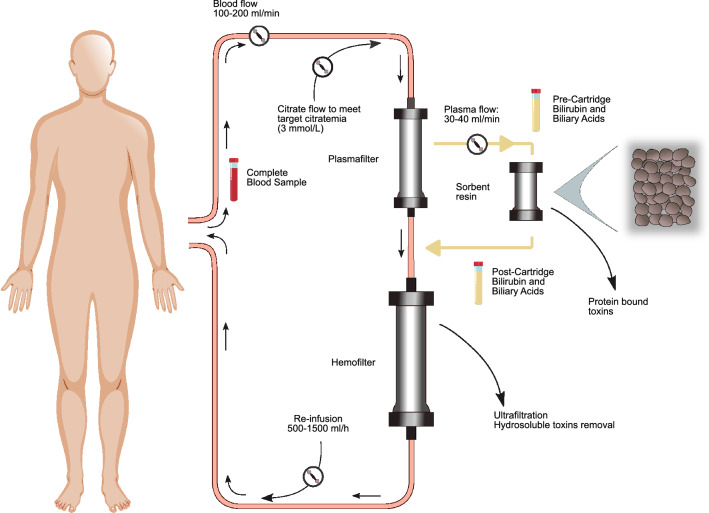
CPFA circuit. The tubes indicate the locations where samples were drawn

Harm et al*.* have demonstrated in vitro that the hydrophobic styrene resin, used in the adsorbent filter of the coupled plasma-filtration and adsorption (CPFA) system, has a high affinity for bilirubin, bile acids, tryptophan and phenols [10]. The CPFA system consists of plasma separation followed by plasma adsorption through a single hydrophobic styrene resin cartridge. The subsequent reconstitution of the purified plasma to the whole blood and the passage through a hemofilter allows further removal of water-soluble toxins (Fig. 1). CPFA is currently used in the treatment of patients suffering from acute kidney injury (AKI) and septic shock, due to its efficacy in the removal of cytokines and inflammatory mediators without significant loss of albumin [11].

Herein, the aim of the present study is to apply the CPFA extracorporeal depurative system to patients suffering from ALF or AoCLF in order to test its purifying capacity on both the main albumin-related toxins and the water-soluble toxins, as well as the biocompatibility of the system.

## Methods

Twelve patients affected by ALF or AoCLF and hospitalized at the Internal Medicine Unit of S. Orsola University Hospital in Bologna, Italy, from June 2013 to November 2017 were enrolled in the present observational, non-randomized study. The inclusion criteria for CPFA treatment were a diagnosis of ALF or AoCLF not responsive to the usual medical therapy (UMT) and a rapidly progressive serum total bilirubin > 20 mg/dL or MELD Score > 20 at the time of enrollment [12, 13]. Rapidly progressive serum bilirubin is defined as > 50% bilirubin increase from the levels recorded at admission. The bilirubin and MELD cut-off values for patient enrollment were arbitrary and based on our previous experience in this field [12, 13]. The UMT included: treatment of acute variceal bleeding with terlipressin or somatostatin together with variceal band ligation; prophylaxis of variceal bleeding performed with the use of beta-blockers and/or variceal band ligation [14]. Ascites was treated with sodium restriction and large-volume paracentesis combined with albumin administration. Aldosterone antagonists were given only if there were no contraindications, such as renal failure or hyperkalemia [15]. Hepatorenal syndrome (HRS) was diagnosed according to the classic criteria of the International Ascites Club and treated initially with vasoconstrictors and albumin [16]. Hepatic encephalopathy was treated with standard therapy, including lactulose or lactitol and cleansing enemas [17]. Spontaneous bacterial peritonitis was treated with third generation cephalosporins [18]. Blood glucose levels were kept under close control; proton pump inhibitors and antiviral therapy were administered when indicated.

The exclusion criteria were: active bleeding with hemoglobin < 7 g/dL in spite of treatment of variceal bleeding and blood transfusions; hypotensive instability (defined as a mean arterial pressure < 60 mmHg despite vasopressor therapy) [19]; acute coronary syndrome in the last month; respiratory failure with the need of mechanical ventilation; acute mental illness preventing patients from cooperating and signing the informed consent.

Patients’ clinical history and data collected from personal electronic medical records were used to calculate the Sequential Organ Failure Assessment score (SOFA) and Model for End-Stage Liver Disease (MELD) [20]. Both SOFA and MELD scores have been assessed before the start and at the end of each CPFA session. The patients’ main characteristics are reported in Table 1.

**Table 1:** Baseline characteristics of patients and main features of CPFA treatment, the results are showed as median and min/max range

Clinical and biochemical baselines features	
Patients (n)	12
Age (years)	51 (33–75)
M/F (n)	10/2
Acute liver failure (n)	3
Acute-on-chronic liver failure (n)	9
SOFA score	6 (4–12)
CLIF-SOFA (score)	9 (9–12)
MELD (score)	23 (20–34)
HE West Haven Criteria (score)	1 (1–3)
WBC (u/μL)	11,650 (4930–37,800)
Hemoglobin (g/dL)	9.1 (7.0–13-7)
Platelet count (u/μL)	225,000 (19,000–421,000)
CRP (g/dL)	3.7 (1.29–12)
aPtt	1.64 (0.91–10)
INR	1.34 (1.17–1.97)
Sodium (mmol/L)	138 (132–144)
Potassium (mmol/L)	3.7 (2.8–4.1)
Fosfate (mg/dL)	2.6 (1.1–4.9)
Creatinine (mg/dL)	0.98 (0.55–5.64)
Urea (mg/dL)	50 (193–9)
Lactate (mmol/L)	1.85 (0.9–4.6)
Total bilirubin (mg/dL)	33.25 (21.5–71.21)
Indirect bilirubin (mg/dL)	7.58 (3.58–23.47)
Direct bilirubin (mg/dL)	24.57 (13.99–54.58)
Biliary acids (μmol/L)	185 (84–208)
AST (U/L)	212.5 (66–534)
ALT (U/L)	137 (24–475)
AP (U/L)	166.5 (115–317)
GGT( U/L)	220 (110–428)
Albumin (g/dL)	2.8 (1.9–3.5)
Urine output (mL/24 h)	1500 (300–2000)
Liver failure cause	
Alcoholic	8
Viral (HBV–HCV–HDV)	2
Post-surgical jaundice	1
Lithiasic cholangitis	1

The min/max range is defined within the brackets

*CVC* central venous catheter, *CLIF-SOFA *chronic liver failure-sequential organ failure assessment

CPFA was performed using the Lynda machine for CRRT (Bellco/MedTronic, Mirandola, Italy) from 2013 to 2014 and with the Amplya machine for CRRT (Bellco/MedTronic, Mirandola, Italy) from 2015 to 2017. The switch from the Lynda to the Amplya machine was carried out after the latter became available on the market the newest and improved system for CRRT and CPFA. The patients were treated with CPFA in the Dialysis ward of the Nephrology, Dialysis and Renal Transplantation Unit at S. Orsola University Hospital in Bologna, Italy. The general care of the patients was provided in the Internal Medicine Unit of the same Hospital.

The CPFA procedure is represented in Fig. 1. Briefly, blood passes through a polyethersulfone plasma filter (Micropes) that separates plasma from whole blood. Plasma than passes through an adsorbent cartridge containing divinylbenzene styrene resin and the purified plasma is returned to the whole blood. The blood then passes through a hemofilter (polyphenylene). The resin cartridge is used to remove albumin-bound toxins from the plasma, while the hemofilter is used to remove water-soluble toxins [6].

Before the CPFA session, the circuit, the membranes and the cartridge were rinsed with 2 L of saline solution (0.9%). The circuit setup lasted 20 min. Anticoagulation of the extracorporeal circuit was obtained with unfractionated heparin (Epsoclar, 25,000 UI/5 mL, Hospira, Napoli) or with calcium-citrate loco-regional anticoagulation. Heparin was administrated with an initial bolus of 50 UI/kg followed by a continuous infusion of 5–10 IU/kg/h; citrate was administered through 5-L-bags in predilution named Bellco Citrachoice 10/2 and the dose achieved in the blood was 3 mmol/L, with a final circuit concentration of 3 mM. Anticoagulation with citrate was employed only with the Amplya machine, to reduce the bleeding risk. Citrate anticoagulation was used after the Ethical Committee of S. Orsola University Hospital approved an amendment to the study protocol (EM/175/0, study protocol no. 87/2012/Disp/O) relating to the use of citrate anticoagulation in patients with liver failure during CPFA. At that time, the Lynda machine was not any longer used for CPFA.

CPFA treatment was performed through a central venous catheter that was placed either in the internal jugular vein or in the femoral vein (Mahurkar^®^, length 19.5–24 cm, diameter 11.5 French, MedTronic, Minneapolis, USA). To reduce the risk of catheter recirculation, the internal jugular central venous catheters were placed with the tip into the upper right atrium, while in the case of femoral catheters, a 24 cm-long catheter was put in place [21]. Patients had intermittent treatments that lasted 6 h. We considered which patients, with and without AKI, required dialysis. AKI has been staged according to the RIFLE criteria [22], and the frequency of treatment was defined on the basis of the clinical and laboratory findings.

CPFA treatment was interrupted in the following situations: (a) liver function recovery in terms of stable bilirubin reduction; (b) liver transplant; (c) a patient meeting one or more of the original exclusion criteria; (d) the maximum of 5 consecutive treatments per patient, required by the study protocol, was reached. After the end of the CPFA treatments, a 12-month follow-up was organized, to evaluate survival.

Blood samples were collected at the start of CPFA (t0) and then every hour until the end of the session (t60, t120, t180, t240, t300 and t 360) to assess bilirubin and bile acids variations. At t0 and t360 we measured hemoglobin, white blood cells, platelets, International Normalized Ratio (INR), activated Partial Thromboplastin Time (aPTT), urea, creatinine, sodium, potassium, calcium, phosphate, alanine aminotransferase (ALT) and aspartate aminotransferase (AST). Blood samples were collected from the arterial line before the plasmafilter. The stopped flow method was applied for 30 s before the blood was drawn, to reduce the risk of access recirculation [23]. At the same time intervals mentioned above, fractionated bilirubin and biliary acids levels were measured in the plasma produced by the plasmafilter before and after the resin cartridge. The rebound effect on bilirubin values was evaluated 24 h after the end of the CPFA session. Laboratory results were not corrected for hemoconcentration because no ultrafiltration was prescribed during CPFA. Laboratory values were obtained through commercially available tests (https://ambo.ausl.bologna.it/metro/som/lum/il-lum-per-i-medici-di-medicina-generale-e-gli-specialisti/lum-standard-di-prodotto-27_2_17.pdf/view. Accessed April 2019), biliary acids were measured using a specific enzyme immunoassay (ELISA, Total Bile Acid Assay Kit™, Diazyme Laboratories, Poway CA, USA). The reduction rate per session (RRs) was calculated using the equation RRs = 100 ×  (1 − C_end_/C_start_), where C_start_ and C_end_ are the concentration of substances at the beginning and at the end of the CPFA procedure, respectively. Continuous monitoring of blood pressure, heart rate and hemoglobin oxygen saturation was performed during each CPFA session.

Continuous variables were reported as mean (standard deviation) where distribution was normal and as median [minimum and maximum or interquartile (IQ) range] when distribution was skewed.

Data comparison was made using paired sample *t* test and ANOVA test for continuous variables with normal distribution, Wilcoxon and Kruskall–Wallis test for variables with non-normal distribution and Fisher for percentage variables. Finally, a linear regression analysis was used to compare pre- and post-CPFA hepatic toxins and RRs. p values < 0.05 were considered statistically significant. The statistical analysis was performed using the statistical package for social sciences (SPSS™ for Windows Software Package, version 9.0.1; Chicago IL, USA). Images were performed using GraphPad Prism™ (version 8 for Apple, GraphPad Software Inc., CA, USA).

The Ethical Committee of S. Orsola University Hospital approved the study protocol (no. 87/2012/O/Disp, ClinicalTrials.gov Identifier: NCT03312036) and each patient signed an informed written consent before taking part in the study.

## Results

**Fig. 2:**
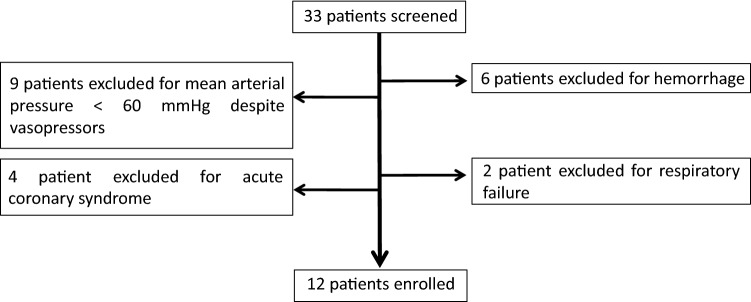
Flow chart of patients screened before the enrollment in the study

Twelve patients (10 males) with a median age of 51 years (range 33–75 years) were enrolled (Fig. 2). ALF was diagnosed in 3 subjects, while AoCLF affected the others. The cause of liver failure was alcoholic in 8 patients (66.6%), viral hepatitis in 2 patients (16.7%), post-surgical cholestasis and lithiasic cholangitis, respectively, in the remaining 2 patients (16.7%). Clinical baseline characteristics are summarized in Table 1. The median age of patients with alcoholic AoCLF was 45 years (range 33–52 years).

A total of 31 CPFA sessions were performed with a median of 2 sessions per patient (range 1–5). All the patients underwent 1 CPFA session, 9/12 had 2 CPFA sessions, 5/12 had 3 CPFA sessions, 3/12 patients had 4 CPFA sessions, 2/12 had 5 CPFA sessions. Blood and plasma volume processed for each CPFA session were 54 L (range 72–41) and 8.2 L (range 3.8–11) respectively and the median ratio between plasma treated volume and patient weight was 0.10 L/kg/session (range 0.04–0.13). The median time between first and last CPFA session was 4.5 days. CPFA treatment features for heparin and citrate patients are provided in Table 2.

**Table 2:** CPFA treatment features for heparin and citrate patients

	Heparin	Citrate	P
Patients (n)	7	5	–
CVC (n, jugular/femoral)	2/5	0/5	–
CPFA sessions (n)	15	16	–
CPFA duration (h)	6	6	–
Blood flow rate (mL/min)	150 (100–200)	150 (100–220)	0.67
Effluent volume (mL/session)	9 (5.1–15)	14.4 (5.1–26.4)	0.12
Fluid removal (mL/session)	0	0	–
Plasma treated (mL/session)	8.4 (6.4–11.5)	8.3 (4.4–11.5)	0.13
Plasma processed dose (L/kg/session)	0.10 (0.17–0.06)	0.10 (0.15–0.04)	0.91
Plasma volumes treated (volumes/session)	2.0 (1.3–3.2)	2.0 (1.0–3.2)	0.26
Heparin amount (IU/session)	4000 (7500–1500)	–	–
Circuit citratemia (mmol/L)	–	3.0	–

The results are showed as median and min/max range. The min/max range is defined within the brackets

*CVC* central venous catheter

CPFA did not affect MELD score in AoCLF (median 25, range 20–34 before CPFA start and 24, range 20–35 at the end of the treatment, p = 0.45).

The median SOFA score of ALF and AoCLF patients was 6 (range 4–12) and remained unaltered after the CPFA treatments.

### Detoxification efficacy

**Fig. 3:**
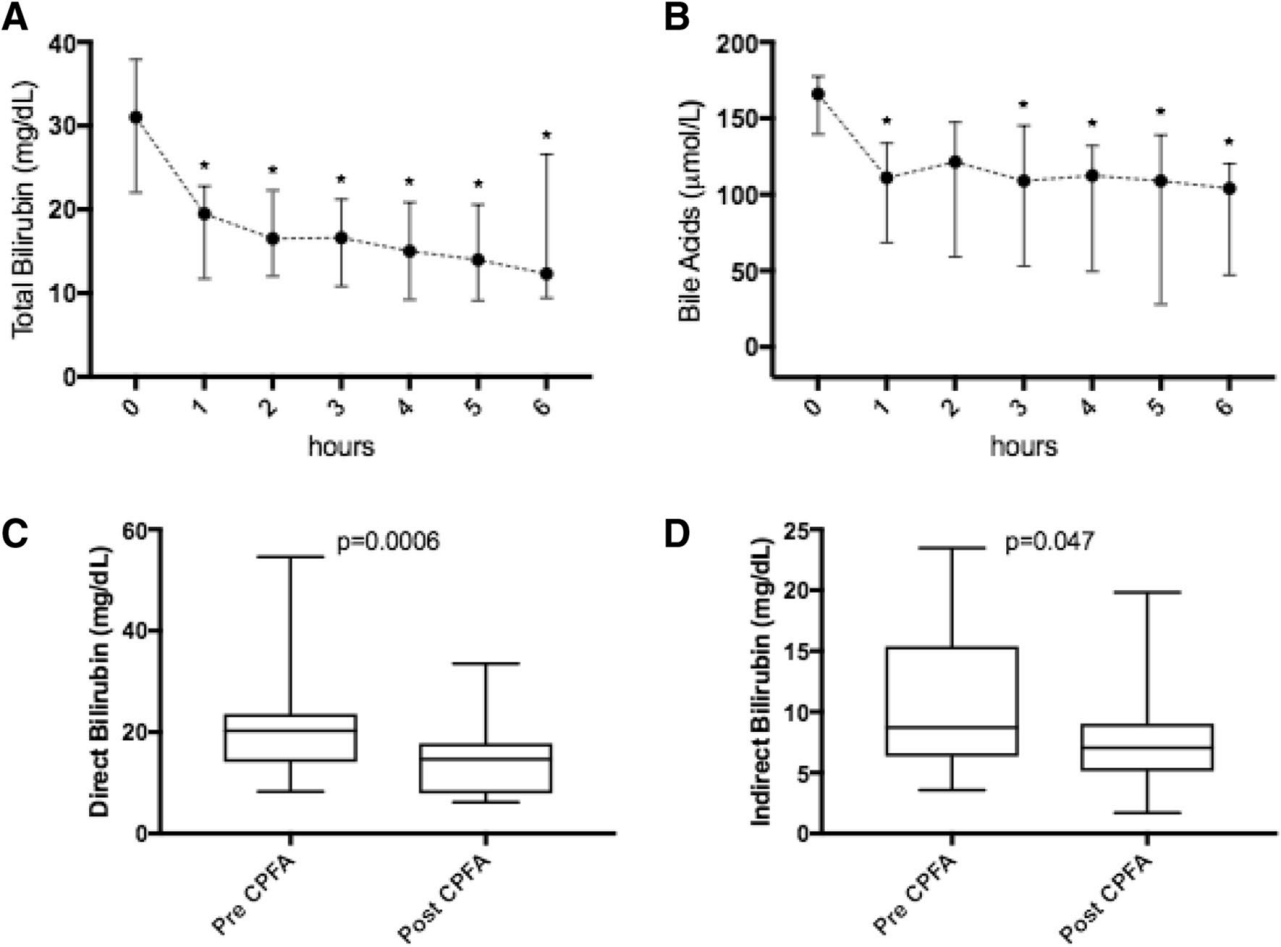
Total bilirubin (**a**) during CPFA sessions, with significance: 0 vs 1 h: p = 0.02; 0 vs 2 h: p = 0.01; 0 vs 3 h: p = 0.01; 0 vs 4 h: p = 0.01; 0 vs 5 h: p = 0.01; 0 vs 6 h: p = 0.04. Bile acids (**b**) during CPFA sessions, with significance: 0 vs 1 h: p = 0.02; 0 vs 2 h: n.s.; 0 vs 3 h: p = 0.03; 0 vs 4 h: p = 0.03; 0 vs 5 h: p = 0.04; 0 vs 6 h: p = 0.01. Direct and indirect bilirubin (**c**, **d**) pre and post-CPFA session. Values are expressed as median with interquartile range and representing 31 CPFA sessions

After a single CPFA session both total and fractionated bilirubin and biliary acids significantly decreased. Total bilirubin and bile acids decline during CPFA is represented in Fig. 3a, b.

**Fig. 4:**
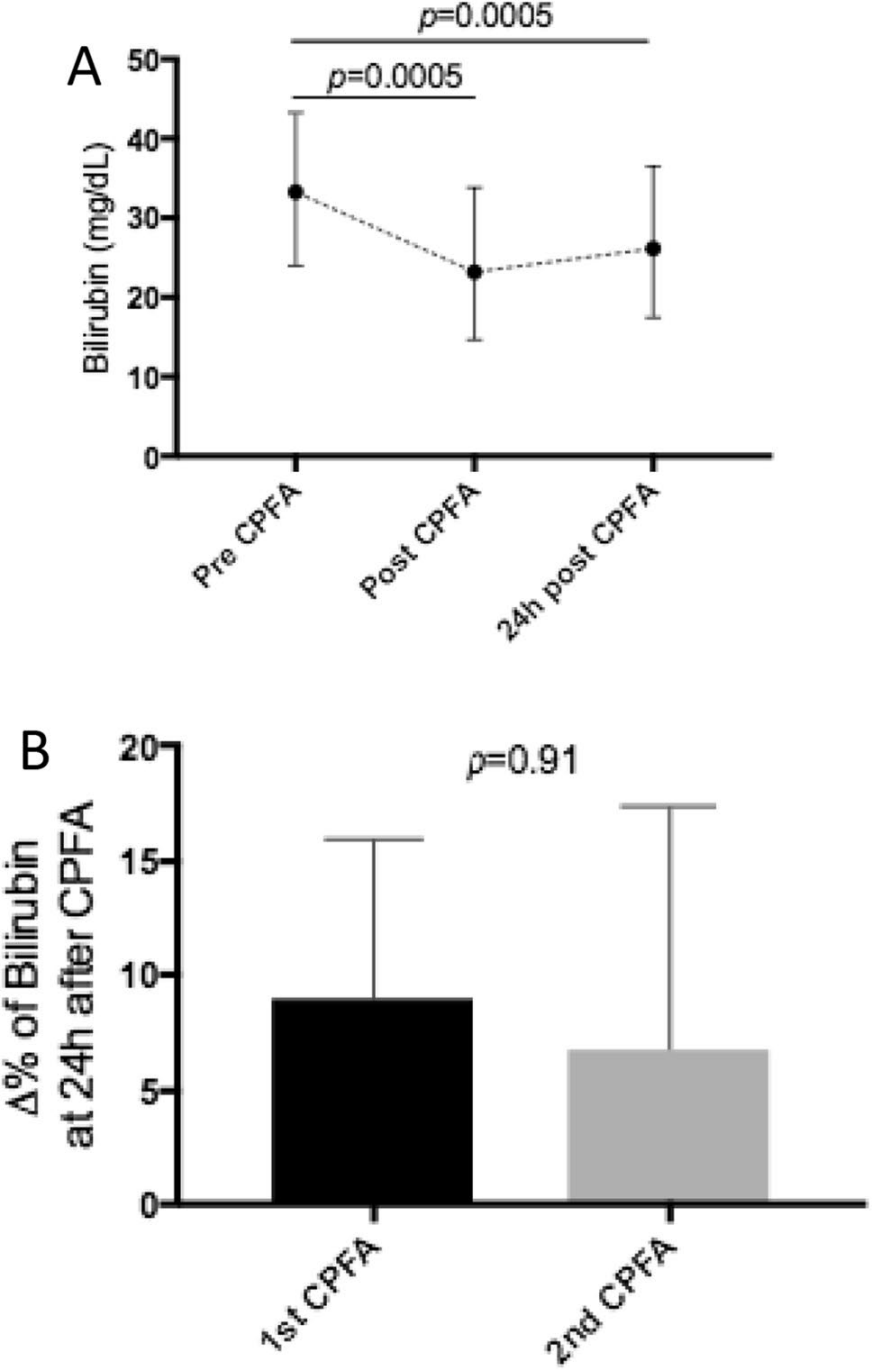
Bilirubin rebound 24 h after the first CPFA session (12 patients) (**a**). Comparison of bilirubin rebound between the first (12 patients) and the second CPFA sessions (9 patients, p = 0.91) (**b**)

**Fig. 5:**
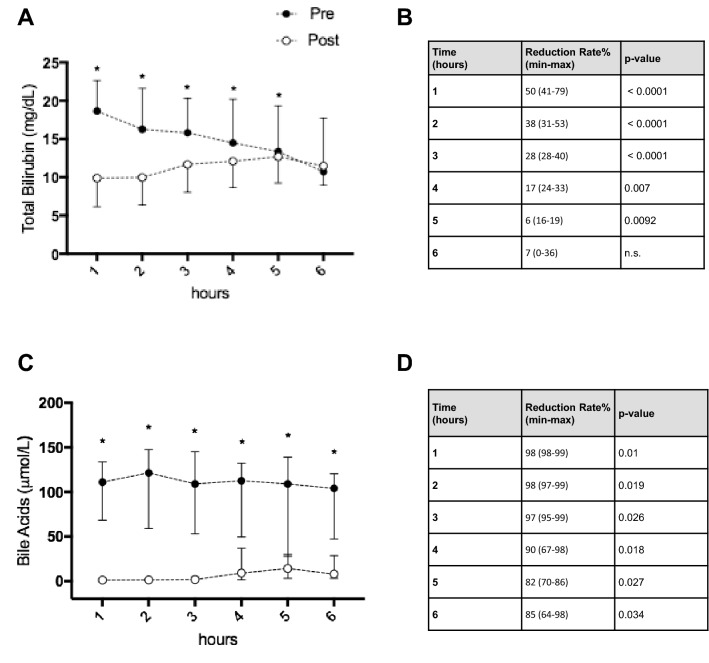
Pre and post-cartridge bilirubin values during 18 CPFA sessions (**a**) and bile acids during four CPFA sessions (**c**), with relative reduction rates (**b** bilirubin, **d** bile acids). Values are expressed as median with interquartile range

Direct and indirect bilirubin blood-levels are showed in Fig. 3c, d. Bilirubin values 24 h after the end of the first CPFA session were significantly lower than the pre-CPFA values, despite the bilirubin rebound (Fig. 4a). After the first CPFA session (12 patients) the median bilirubin rebound was 8.9% (IQ range 2.9–15.5). After the second CPFA session (9 patients) the median bilirubin rebound was 6.8% (IQ range 2.0–16.8). As for the bilirubin rebound rate, no differences were found between the first and the second CPFA sessions, p = ns (Fig. 4b). In a subgroup of 6 patients, for a total of 18 CPFA sessions, the adsorptive capacity of the resin cartridge, in terms of bilirubin removal, was evaluated as reported in Fig. 5a, b.

In 4 patients, pre-and post-cartridge biliary acid plasma levels were also assessed during 4 CPFA sessions (Fig. 5c, d).

**Fig. 6:**
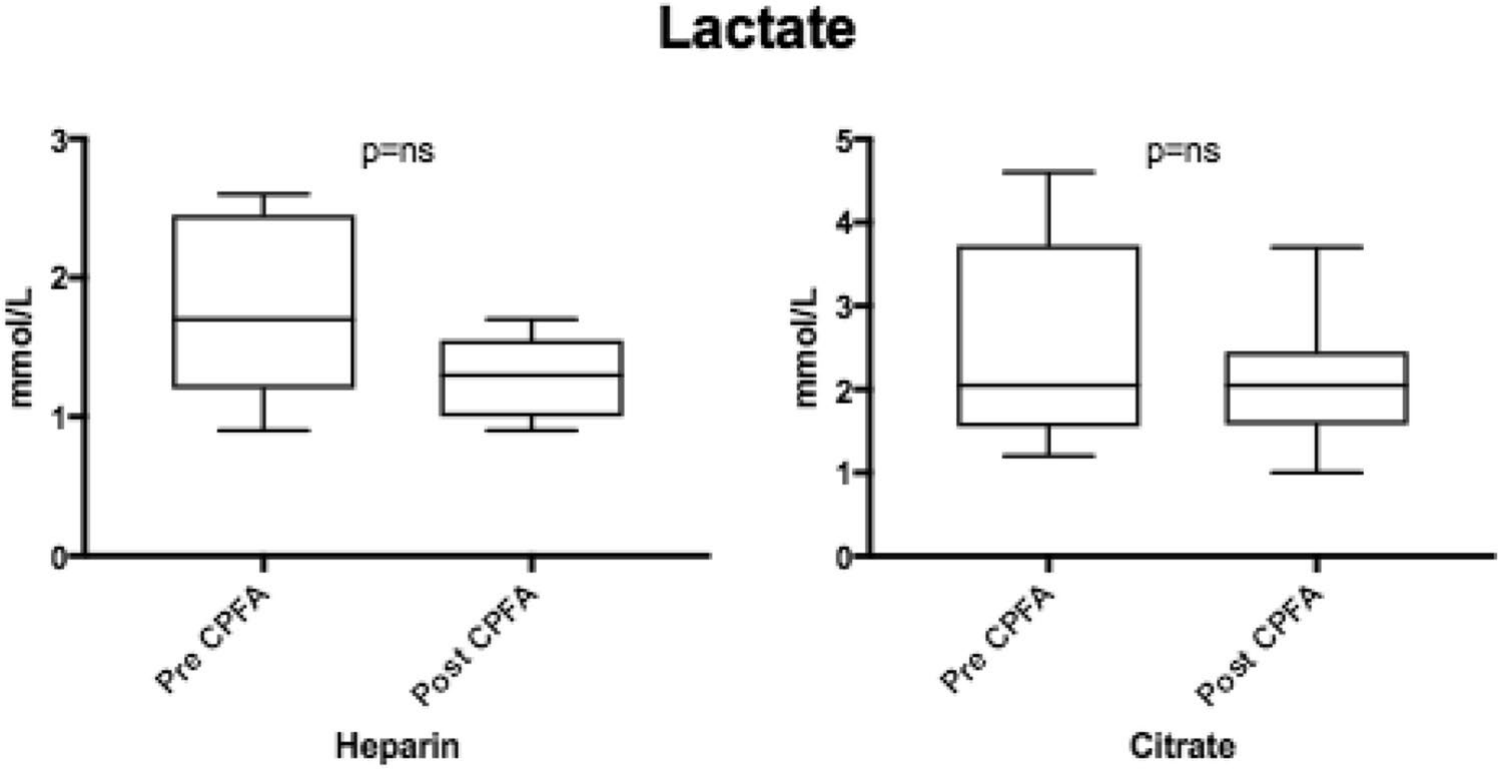
Pre and post-CPFA lactate blood levels in patients treated with heparin (15 CPFA sessions included) or with citrate (16 CPFA sessions included)

According to the RIFLE criteria, 4 patients developed AKI class I. One patient developed hepatorenal syndrome and AKI Class F (Failure), with serum creatinine and urea levels up to 5.64 mg/dL and 194 mg/dL respectively. He recovered normal renal function after CPFA treatment and standard medical therapy: a 6-h-long CPFA was administered daily for 4 days and an effluent volume of 20 L per session was obtained. In the other patients, the concentration of water-soluble toxins urea and creatinine did not significantly change pre- and post-CPFA. Median creatinine and urea pre-CPFA were respectively 0.98 mg/dL (range 0.44–5.00) and 50 mg/dL (range 9–193), while median post-CPFA levels were respectively 0.87 mg/dL (range 0.39–3.86) and 35 mg/dL (range 8–145), p = 0.45. Lactate blood levels did not show a significant decrease (Fig. 6), particularly in subjects with citrate-based circuit anticoagulation (1.7 mmol/L vs. 1.2 mmol/L, p = ns).

### Sepsis

Sepsis, defined according to the criteria of the Third International Consensus Definitions for Sepsis and Septic Shock [24], was reported in seven patients. Sepsis was due to pulmonary infection in three cases and spontaneous bacterial peritonitis in 4 cases. In all patients with sepsis a + 2 SOFA sub-score for respiratory system and liver was reported. Four patients were treated with CPFA and heparin, 3 with CPFA and citrate. Among patients affected by sepsis, a total of 23 CPFA treatments were performed: 10 sessions were carried out using heparin as anticoagulation and 13 using citrate. One patient with sepsis of pulmonary origin died during the follow-up period because of infection-related complications.

### Hemodynamic tolerance

CPFA treatment was hemodynamically well tolerated. Median value of mean arterial pressure (MAP) remained stable during the CPFA session: 91.5 mmHg, 90.0 mmHg, 88.0 mmHg, 89.9 mmHg, 79.2 mmHg, 82.4 mmHg, 94.5 mmHg at t0, t60, t120, t180, t240, t300, t360 respectively (p > 0.05). Heart rate resulted stable during CPFA treatment. Vasoactive drugs were not administered to any patient.

### Safety

All treatments were well tolerated. Safety was evaluated measuring several biochemical parameters in the serum: hemoglobin, white blood cells count, platelets, sodium, potassium and coagulation parameters variation before and after the CPFA session.

Only coagulation parameters differed significantly: INR and aPTT rose from t0 to t360 in the whole population (1.34 vs 1.69, and 1.64 vs 2.81 respectively p < 0.001). Nonetheless, no hemorrhage was reported after CPFA. Similar results were reported for patients treated with heparin or regional citrate anticoagulation: in the first group, median INR was 1.62 at t0 and 2.0 at t360 (p < 0.001), while, in the second group, values were 1.3 at t0 and 1.5 at t360 (p < 0.001). Median aPTT increased from 1.64 (t0) to 2.81 (t360) in the whole population. Regarding the heparin group, aPTT was 1.8 at t0 and 3.48 at t360 while in the citrate group aPTT rose from 1.81 (t0) to 2.12 (t360) (p < 0.001). The restoration of normal values 24 h after the end of CPFA treatment was obtained.

### Patient survival

In this study 9/12 patients (75%) completed 1 year of follow-up: 8 recovered their basal liver function and 1 patient received an OLT. Three patients died: 2 during hospitalization and 1 at 4 months of follow-up for an acute cardiac event.

## Discussion

HERCOLE is a prospective, observational, open-label, single-center clinical trial aimed at investigating the depurative efficacy and safety of CPFA treatment in 12 patients affected by ALF or AoCLF. The dose of plasma treated in this instance was 0.1 L/kg (sessions lasting no longer than 6 h) while doses of 0.2 L/kg/die are reported in the literature in case of septic-shock or AKI (without ALF or AoCLF) [11].

Bilirubin, which represented the main marker for estimating hepatic function, decreased significantly with CPFA. The RRs of total bilirubin was 30% while the RRs for indirect bilirubin was 24%. The pre- and post-bilirubin values were obtained for each CPFA session. Nonetheless, in 5/31 CPFA sessions, serum indirect bilirubin increased from 2 to 11%, despite the concomitant reduction of direct bilirubin. This phenomenon can be explained considering direct bilirubin’s faster dissociation rate from albumin: as a consequence, the rate of indirect bilirubin raises and overcomes the levels of direct bilirubin. Unfortunately, pre- vs post- resin sampling was available only for 18/31 sessions.

The results of this study lead to three main considerations: (a) resin cartridge is effective in bilirubin removal, (b) cartridge saturation is a progressive phenomenon and peaks at the 6th hour, (c) the adsorbing ability of the resin is maintained when total bilirubin is ≥ 20 mg/dL. When bilirubin is higher than 50 mg/dL, early resin saturation is reported. Such conditions require either a daily CPFA treatment, resin replacement after 3 h, or an increase in whole amount of resin used [25]. The amount of additional resin depends on resin saturation times and needs further investigation. Resin saturation can be diagnosed when the post-cartridge bilirubin value resembles the pre-cartridge value. If, for example, this occurs after 3 h of CPFA during a 6-h length session, for the next CPFA session we would need a cartridge with double the amount of resin.

On the other hand, whenever serum total bilirubin levels were ≥ 20 mg/dL at the start of treatment and < 20 mg/dL at the beginning of the following CPFA sessions, only partial resin cartridge saturation was observed and higher bilirubin RRs were recorded. Therefore, in this condition, CPFA session length may be increased with the purpose of allowing complete cartridge saturation.

Bilirubin removal dynamics are further complicated by the slow equilibration of bilirubin between the intravascular and extravascular pool, were the concurrent bilirubin production takes place: this is the so-called “rebound phenomenon”. In the present study, it was evaluated after 24 h from the end of the CPFA session. When considering the first CPFA session, the bilirubin rebound did not differ significantly from the values at the end of CPFA. The rebound was similar in those patients that underwent further CPFA sessions, but not in those who did not require any more CPFA. In these cases, we can speculate that the amount of bilirubin stored within the extravascular pool gradually diminished and the serum bilirubin level gradually fell as well. The rebound phenomenon after MARS was firstly observed by Covic et al. in 2003 [26]. The authors described six children affected by liver failure from mushroom poisoning. After 24 h from the first MARS session, all the patients showed a bilirubin rebound of + 39%; 24 h after the second MARS session, only 2/6 cases showed a bilirubin rebound of + 220%. Chiu et al., in 2006, retrospectively evaluated bilirubin rebound after MARS treatment in a cohort of 22 patients who underwent 72 6-h long MARS sessions. They considered the rebound as a constant phenomenon; its mean value was 16.8% 24 h after the first MARS session [27].

Bile acids are weakly bound to albumin, as suggested by the higher dissociation rate compared to bilirubin. A significant decrease of bile acids was obtained with CPFA. The median RRs for total biliary acids in the present study was 30%. The pre- and post- biliary acids values were obtained for each CPFA session. The comparison of biliary acids values pre- and post-cartridge demonstrated an impressive decrease, testifying a great affinity of the resin for such molecules. Despite the high removal rate, resin saturation was not observed—allowing for the hypothesis that longer CPFA sessions could be effective for further bile acid removal. Furthermore, after the 1st hour of treatment, plasma pre-cartridge concentration did not decrease, suggesting the presence of a rebound phenomenon. However, pre- and post- resin sampling was performed on a small sub-group of patients and a bigger sample is needed for further investigation.

In CPFA, the clearance of water-soluble toxins, such as urea, creatinine and lactate, occurs in the hemofilter, downstream of the adsorbent cartridge, and is regulated by the convective volumes achieved. In our study, during CPFA, reinfusion occurred in post-dilution whenever heparin was administered as an anticoagulant, and both in pre- and post-dilution when citrate anticoagulation was used instead. The mean convective volume per single CPFA session was 9 L when the anticoagulation was obtained with heparin, while it was 11.5 L in citrate-treated patients. In citrate treatment, the convective volume was measured considering that the depurative efficacy of pre-dilution is about 50% of the post-dilution mode. In our experience, the removal of urea and creatinine was not very efficient (RRs of 20 and 22% respectively). This may be attributed to one of the following facts: (1) a relevant number of treated subjects (7/12) had normal renal function; (2) a low convective volume was prescribed; (3) diffusive clearance is not allowed by the present CPFA system. The most relevant hydrosoluble toxin evaluated during our study was lactate. An increase of serum lactate may be observed in patients with ALF or AoCLF due to the reduced hepatic clearance. Sepsis occurs frequently in this type of patients, with consequent increase of lactate: tissue hypoxia induces pyruvic acid accumulation, due to the reduced efficiency of the Krebs cycle. Then, lactate dehydrogenase turns pyruvic acid into lactate, which increases in blood. In these cases, especially in patients with lactate acid > 3.4 mmol/L [28, 29], citrate anticoagulation has to be provided with caution during extracorporeal detoxification. The reduced efficiency of the Krebs cycle reduces the metabolism of citrate, and a further increase of serum lactate levels may occur. In the present study, 23/31 CPFA sessions were performed in seven septic patients with liver failure. Sepsis may have increased lactate levels in patients with ALF or AoCLF. Nonetheless CPFA treatments were not associated with a significant overall reduction of lactate levels when heparin or citrate anticoagulation was used. It seems that lactate detoxification is particularly impaired in patients with simultaneous sepsis and liver failure. Indeed, no significant serum lactate accumulation (> 5 mmol/L), metabolic acidosis, hypocalcaemia or increased total calcium/ionized calcium ratio were reported in patients treated with citrate. Only one patient started CPFA with lactate levels > 3.4 mmol/L during five consecutive CPFA sessions. In 1/5 CPFA sessions the final values of lactate exceeded 3.4 mmol/L and no toxicity occurred. In addition, recent data points at the effectiveness and safety of CPFA in sepsis, when it is restrained in well-defined clinical settings [30]. Mariano et al. for example reported an increased survival rate in 39 severe burns patients with septic shock-associated AKI who were treated with CPFA [30].

In case of AKI requiring dialysis, or when a high lactate clearance is needed, a higher convective volume has to be scheduled as a conventional CRRT requires [31]. It can be obtained by: (1) increasing the CPFA session length; (2) using a more concentrated citrate bag in the predilution mode during CPFA (Bellco Citrachoice 20/4) in order to obtain the same dose of citrate in blood (3 mmol/L) but allowing the increase of the post dilution fluid infusion.

Despite its depuration efficacy, CPFA did not reduce MELD score in our cohort. This may be explained by the rise in median INR both in patients treated with heparin, and with regional citrate.

During our study, all treatments were hemodynamically well tolerated, and no major adverse events were reported. In particular, there were no episodes of hypotension and/or intra-treatment arrhythmias. Hemodynamic tolerance is intrinsic to the depurative technique proposed: (a) during CPFA no albumin leakage occurs, while hemofiltration stabilizes plasma osmolarity and decreases the body core temperature resulting in a better preservation of blood pressure [32]; (b) the priming volume of the extracorporeal circuit does not exceed the volume of 250 mL. The analysis of survival does not allow for definitive conclusions, mainly due to the limited number of patients and the lack of a control group.

Other adsorption columns containing styrene divinylbenzene copolymer are available on the market and seem to achieve similar results to those obtained in the present study. Nonetheless, this is the first observational study to consider in vivo the removal of protein-bound and non-protein-bound toxins during liver failure with the CPFA resin, making it different from all those available until now. The resin, namely CG 300c, is a neutral macroporous styrene divinylbenzene copolymer; its beads have a mean diameter of about 100 μm and a pore size of 30 nm [10]. The amount of resin into the CPFA Mediasorb column is 70 g and allows an adsorbing surface area of 50,000 m^2^_._ The principal competitors of CPFA are:

1. MARS therapy (Baxter), which uses an albumin dialysate regenerated by a low flux dialysis filter, by a charcoal cartridge and by an anion exchange resin. No data were available about the chemical characteristics of this resin [13].
2. Prometheus (Fresenius Medical Care) that works by means of fractionated plasma separation and adsorption. Two cartridges containing styrene divinylbenzene copolymer allow plasma adsorption. The first one is a neutral resin; the second one is an anion exchange resin. Each cartridge has an amount of 350-g resin, the resin beads have a mean diameter of 600 μm, the pore size is 8–9 nm, the adsorbing surface area is 120,000 m^2^ [33].
3. Cytosorb cartridge (Medasorb Technologies) that is used for hemoadsorption. It consists of styrene divinylbenzene beads with a diameter of 450 μm; the pore diameter is between 0.8 and 5 nm, the surface area is 850 m^2^/g, the amount of resin is 300 g [10].
4. Plasorba BR-350 (Asahi Medical) is a cartridge filled of an anion exchange resin. The resin consists of 350-g styrene divinylbenzene copolymer [34]. The resin beads diameter is 400 μm.

Every resin has different characteristics and is coupled with different techniques in extracorporeal detoxification (albumin dialysis, plasma separation and adsorption, hemoadsorption). But some other differences can be found. Firstly, the cost: CPFA costs 1.250 euros, MARS and Prometheus 3.000 euros; Cytosorb 1.600 euros; Plasorba 900 euros. Secondly, CPFA allows also CRRT while MARS, Cytosorb and Plasorba need to be combined to a CRRT system.

## Conclusions

Treatment with CPFA has been shown to be effective in removing bilirubin and bile acids in liver failure patients with a good safety profile, although it is a complex system in terms of technical application. The small number of patients and their rather inhomogeneous clinical presentations are a weakness of this study. It is worth noting that this mainly depended from the setting of the CPFA treatment, which was the dialysis ward. Most of the patients screened, in fact, were admitted to the intensive care unit, were the CPFA was not carried out. A second weakness of the study is the variability of the parameters taken into consideration. This can be explained by the fact that rapidly progressive bilirubin was as inclusion criteria and thus, in many cases, the nephrologist was alerted when bilirubin values were much higher than 20 mg/dL. To date is not possible to consider CPFA as an “artificial liver”. Nonetheless, a randomized controlled trial with an adequate number of patients and an adequate panel of observed toxins is needed to define if CPFA can be used more effectively to achieve blood purification in patients affected by ALF or AoCLF.

## Data Availability

All published data are available at our unit, additional data will be provided if requested.
